# Prevalence and genotypic distribution of high-risk human papillomavirus (HPV) among ever-married women in coastal regions of Bangladesh

**DOI:** 10.1371/journal.pone.0313396

**Published:** 2024-12-12

**Authors:** Snigdha Chakraborty, Ashrafun Nessa, Noor-E Ferdous, Mohammad Mosiur Rahman, Mohammad Harun Ur Rashid, Asma Akter Sonia, Md Foyjul Islam

**Affiliations:** 1 Department of Gynaecological Oncology, BSMMU, Dhaka, Bangladesh; 2 Department of Pathology, BSMMU, Dhaka, Bangladesh; 3 Directorate General of Health Services, Dhaka, Bangladesh; 4 National Center for Cervical and Breast Cancer Screening and Training, BSMMU, Dhaka, Bangladesh; 5 FETP,B Fellow (advanced), Institute of Epidemiology, Disease Control and Research (IEDCR), Dhaka, Bangladesh; UFRN: Universidade Federal do Rio Grande do Norte, BRAZIL

## Abstract

**Background:**

Understanding the distribution of type specific human papillomavirus (HPV) genotypes in screen-detected lesions is crucial to differentiate women who are at a higher risk of developing cervical cancer. This study aimed to find out high-risk HPV genotype distribution among women of the coastal districts of Bangladesh.

**Methods:**

This cross-sectional study conducted from January 2023 to December 2023 aimed to investigate the prevalence and distribution patterns of high-risk HPV genotypes among ever-married women aged 30–60 years residing in three coastal districts of Bangladesh. Sampling was purposive, with 300 participants per district. Exclusion criteria included prior cervical precancer or cancer treatment, hysterectomy, cervical amputation, and pregnancy. HPV DNA specimens were collected and tested using Cobas 4800. Positive cases underwent further genotype analysis with GenoFlowTM HPV Array Test Kit. Statistical analysis utilized SPSS version 25.0, employing Chi-square and Fisher’s Exact tests.

**Results:**

Among 900 participants HR-HPV prevalence was 2.56%. HPV 16 was the most prevalent genotype (38.46%), followed by HPV 66 and HPV 68 (11.54% each). Single infections of HPV 16 predominated (39.13%), while for co-infections HPV 66 and HPV 68 were most common (13.04%). HR-HPV positivity increased with age, peaking at 5.5% in the 55–60 years’ age group. Participants education level, occupation, income, and reproductive history showed no significant association with HPV positivity. District-wise prevalence varied insignificantly, with Jhalokathi exhibiting the highest (3.0%), followed by Cox’s Bazar (2.7%), and Bagerhat (2.0%). HPV 16 was the predominant genotype across districts, with Cox’s Bazar and Jhalokathi demonstrating greater genotype diversity than Bagerhat.

**Conclusion:**

The study concludes that among ever-married women in the coastal districts of Bangladesh, there is a low prevalence of high-risk HPV. The predominant high-risk HPV genotypes identified were HPV 16, followed by HPV 66 and 68. These findings hold significant implications for policy makers, providing guidance for targeted screening strategies and vaccination programs.

## Introduction

Human Papilloma Virus (HPV) is the primary cause of cervical cancer (CC), and over 98% of CC worldwide contained HPV DNA [1]. CC is the fourth most common cancer in women worldwide, with approximately 660,000 new cases and the 350,000 deaths resulting from cervical cancer annually and 94% of them occurred in low-and middle-income countries [2]. The highest rates of both cervical cancer incidence and mortality were found in sub-Saharan Africa, Central America, and South-East Asia [3]. CC is the second most prevalent cancer among Bangladeshi women and constitutes 12% of female cancer. There were approximately 8,268 new cervical cancer cases (10.6 per 100,000 women) and 5.214 deaths (7.1 per 100,00 women) in the year 2018 [3, 4]. A single lifetime screening test might significantly reduce the incidence of CC and subsequent mortality [5]. The Government of Bangladesh adopted visual inspection of cervix with acetic acid (VIA) method for cervical cancer screening in about 600 VIA and Clinical Breast Examination (CBE) centres at primary, secondary and tertiary hospitals of 64 districts of Bangladesh for the women of 30–60 years of age without any cost [6, 7]. However, the low specificity of VIA is an issue of concern and it is entirely a provider dependent test; quality training of paramedical andservice providers are important concerns [8]. Most HPVs typically induce asymptomatic and subclinical infections unless there is immune system compromised. To date, the genomes of nearly 450 distinct HPV types have been isolated and sequenced [9]. Although among the infected women less than 10% get persistent infection, and the persistent infection with a high-risk HPV (HR-HPV) leads to development of cervical cancer [10–12]. Almost all cases of CC and its precursor lesions, cervical intraepithelial neoplasia grade 2 (CIN2) and grade 3 (CIN3), result from the persistent infection of approximately 15 HR-HPV genotypes [13]. Among these genotypes, HPV 16 and HPV 18 account for around 70% of global CC cases, with HPV 16 causing approximately 55–60% and HPV 18 causing 10–15% [14]. Fourteen HPV genotypes (HPV 16, 18, 31, 33, 35, 39, 45, 51, 52, 56, 58, 59, 66, and 68) are considered pathogenic or “high-risk” for causing the development of CC [15, 16]. Recently HPV DNA test has been established as primary screening test for CC [17]. WHO in recent statement as part of 90:70:90 targets for CC by 2030; vaccinating 90% of girls by age 15, screening 70% of women by ages 35 and 45, and treating 90% of women with cervical disease has recommended high precision test for CC screening [18]. HPV DNA test perfectly fits the WHO requirements. As HPV subtypes has different carcinogenic potential, genotyping is required for the triage of HPV-positive women. Apart from HPV16 and HPV18, other HR-HPV types must be accurately known. Therefore, the information regarding the type-specific distribution in a given population is necessary for the evaluation and implementation of appropriate HPV screening tests and prophylactic HPV vaccines. In a developing country like Bangladesh, exploration of alternative modes of screening like HR-HPV test in stead of VIA-based screening and introduction of HPV vaccination is very important to get most effective and feasible prevention method for the country. This study aimed to find out the HR-HPV genotype distribution among ever-married women (women who have been married at least once in their lifetime, regardless of their current marital status) in coastal areas, who represent a neglected population living in hard-to-reach regions with limited access to healthcare services.

## Materials and methods

### Study population and sampling

**Fig 1:**
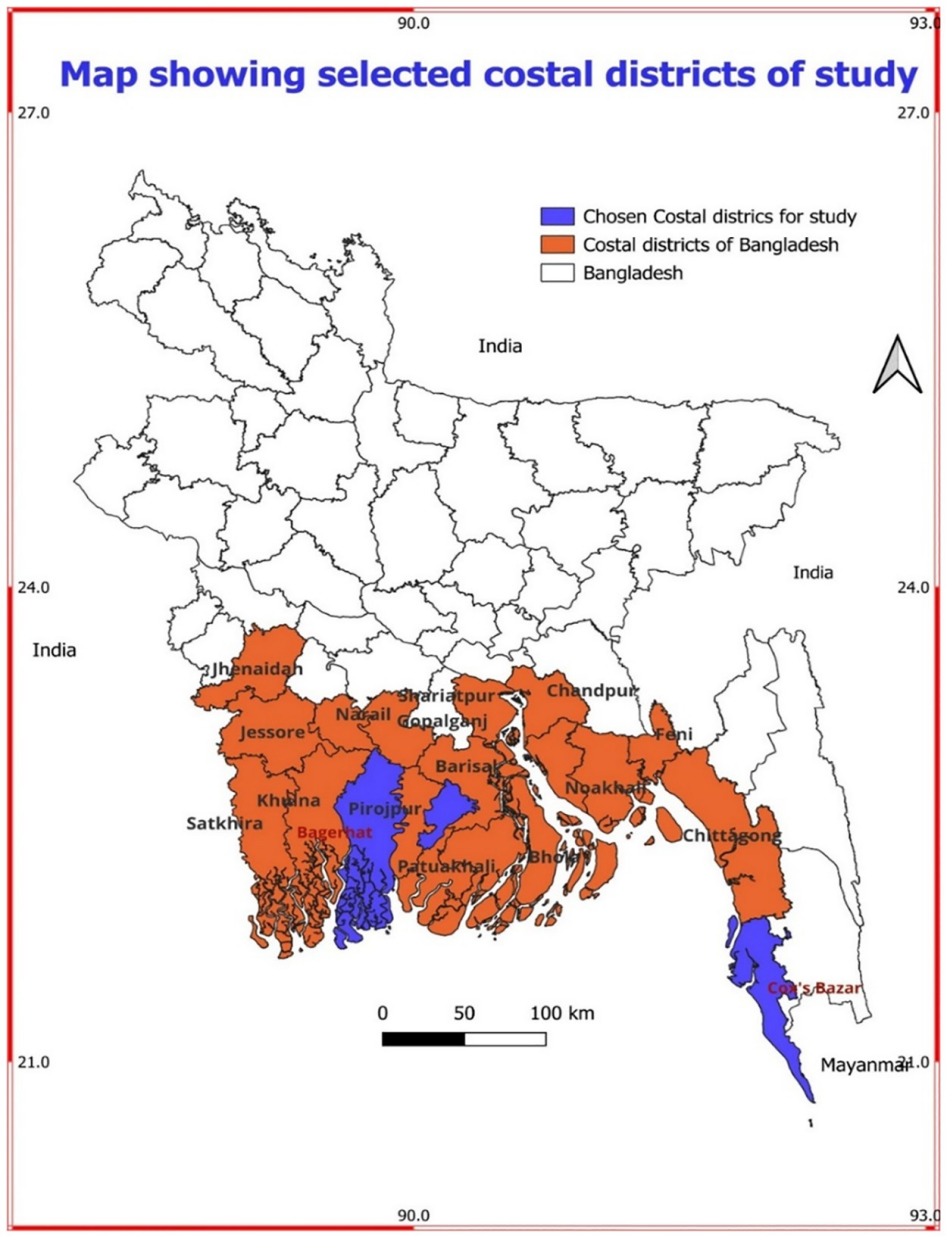
Map showing selected costal districts chosen for study. Map created using QGIS 3.34. Administrative boundaries for districts of Bangladesh provided by GADM, version 4.1, under a Creative Commons Attribution license (https://gadm.org).

This cross-sectional study was carried out from the National Centre of Cervical and Breast Cancer Screening and Training at BSMMU from January 2023 to December 2023 in three randomly selected coastal districts out of 19 costal districts in Bangladesh [Fig 1].

The study selected 900 ever-married women aged 30–60 years using purposive sampling. This sample size was determined through a power analysis aiming for 80% power (β = 0.20) and a 95% confidence level (α = 0.05) to detect a medium effect size of 0.5. The sample size calculation, based on an expected HR-HPV prevalence of 4.2% and a margin of error of 5%, indicated a requirement of approximately 62 participants per site [19]. To ensure robust representation and account for design effects, the total sample was set at 900, with 300 women selected from each of the three districts. and contributed 300 women from each district. Ever married women, attending VIA and CBE screening center of district hospital (DH) attending from surrounding areas were recruited for data collection after counselling. A female research assistant/ trained Senior Staff Nurses (SSNs) provided them detailed information about the study background and objectives, and checked the eligibility criteria before interview. A written informed consent was obtained from each participant. Women with previous treatment due to cervical precancer and cancer, with menstruation at presentation, hysterectomy, cervical amputation and pregnancy were excluded.

A gynaecological examination was performed along with introduction of Cuscos vaginal speculum. HPV DNA specimens were collected prior to VIA test using endo cervical brush by trained doctors/ nurses and placed in ThinPrep (Hologic, Marlborough, MA, USA) fixative liquid medium PreservCyt^™^ solution (20ml) which was validated with the Cobas HPV test following the manufacturer ‘s instruction for collecting cervical specimens. Specimens were transported as soon as possible (within 14 days after collection) to the virology laboratory of BSMMU maintaining appropriate temperature (between 5 to 30°C). A portion t of the specimen (400_μ_l) was promptly subjected to HR-HPV testing with partial genotype analysis (Cobas 4800), while remaining sample kept at 4°C for further use if deemed necessary. The initial HR-HPV test using Cobas 4800 was designed to quickly identify the presence of 14 high-risk genotypes, which are the most clinically relevant. Positive samples were then subjected to further analysis to identify an additional 19 genotypes ensuring comprehensive genotype distribution data. This research was conducted after obtaining ethical clearance from the Institutional Review Board of BSMMU, Dhaka with registration number 764 and memo no (BSMMU/2022/10159).

### HPV DNA detection and genotyping

The HPV DNA detection and genotyping was performed in two phases.

**Initially,** a fully automated real-time PCR amplification and detection analyzer was used to perform the HR-HPV test and its genotype (Cobas 4800, Roche Diagnostics, GmbH, and Mannheim, Germany). This test amplifies target DNA in cervical epithelial cells by polymerase chain reaction (PCR) and nucleic acid hybridization to detect 14 HR HPV types. This assay allows specific identification of HPV types 16 and 18, and pooled detection of HPV types 31, 33, 35, 39, 45, 51, 52, 56, 58, 59, 66, and 68 [20, 21]. The human beta-globulin-oriented fluorescent probe provided quality control of the whole reaction.

**Subsequently,** the HPV positive cases detected initially underwent further genotype analysis using the remaining specimen. *GenoFlow HPV Array Test Kit* was used for in vitro diagnosis [22, 23]. This product is designed for identifying types of HPV using PCR and “Flow-through” hybridization technology. The HPV genomic DNA is amplified by biotinylated primers using PCR. The amplicons are subsequently hybridized to specific capturing probes via “Flow-through” hybridization. Hybridization is then followed by a stringent wash and signal development. This kit can detect the presence of 33 HPV types (HPV types 6, 11, 16, 18, 26, 31, 33, 35, 39, 40, 42, 43, 44, 45, 51, 52, 53, 54, 55, 56, 57, 58, 59, 61, 66, 68, 70, 71, 72, 73, 81, 82 and 84) in cervical samples. HPV-positive samples were classified as either single infections, characterized by the presence of a single genotype, or multiple infections, characterized by the presence of more than one genotype.

### Statistical analysis

Data were collected and managed using Microsoft Excel (version 2016). Statistical analysis was performed using IBM SPSS Statistics, version 25.0 (IBM Corp., Armonk, NY, USA). Nominal data were summarized using frequency and percentage, while continuous data were presented as median and interquartile range (IQR). Chi-square and Fisher’s Exact tests were used to compare categorical variables, with significance set at p<0.05. The prevalence of any HPV infection was defined as the proportion of women testing positive for any HPV genotype via PCR analysis.

## Results

### HR-HPV prevalence and age distribution

The study consisted of 900 women aged between 30 and 60 years with a median age 40 (IQR:35–46). Among the participants, approximately one-fourth of the women fell within the 35–39 years’ age group (25.9%), while the 55–60 years’ age group had the lowest representation (6.1%).". Among the women who underwent testing, 23 individuals (2.56%) tested positive for HR-HPV, while the remaining 97.4% tested negative for these HR-HPV genotypes (Table 1).

**Table 1:** Association of socio-demographic variables with HR-HPV result of study participant (n = 900).

Variables	HR-HPV Positive N(%)	HR-HPV Negative N(%)	p-value*
**Age (years)**			
30–34	2(1.1)	182(98.9)	0.40*
35–39	5(2.1)	228(97.9)	
40–44	6(3.0)	195 (97.0)	
45–49	5(4.1)	117(95.9)	
50–54	2(1.9)	103(98.1)	
55–60	3(5.5)	52(94.5)	
**Participants Educational Status**			
No formal Education	5(4.0)	121(96.0)	0.41**
Primary Education	12(2.8)	421(97.2)	
Secondary Education	5(2.8)	171(97.2)	
Higher Secondary	1(1.4)	72(97.3)	
Graduation and Above	0(0)	91(100)	
**Husbands Educational Status**			
No formal Education	7(5.6)	118(94.4)	0.12*
Primary Education	8(2.3)	343(97.7)	
Secondary Education	4(2.4)	162(97.6)	
Higher Secondary	3(3.0)	98(97.0)	
Graduation and Above	1(0.6)	156(99.4)	
**Occupation of Participant**			
Housewife	22(2.8)	777(97.2)	0.29*
Service Holder	1(1.0)	100(99.0)	
**Monthly Family Income (BDT)**			
≤5000	4(3.6)	106(96.4)	0.28**
50001–10000	12(3.8)	305(96.2)	
10001–20000	5(1.7)	285(98.3)	
20001–50000	2(1.4)	143(98.6)	
≥50000	0(0)	38(100)	

Chi-square test = *, Fisher’s Exact tests = **

### HR-HPV genotype distribution

**Fig 2:**
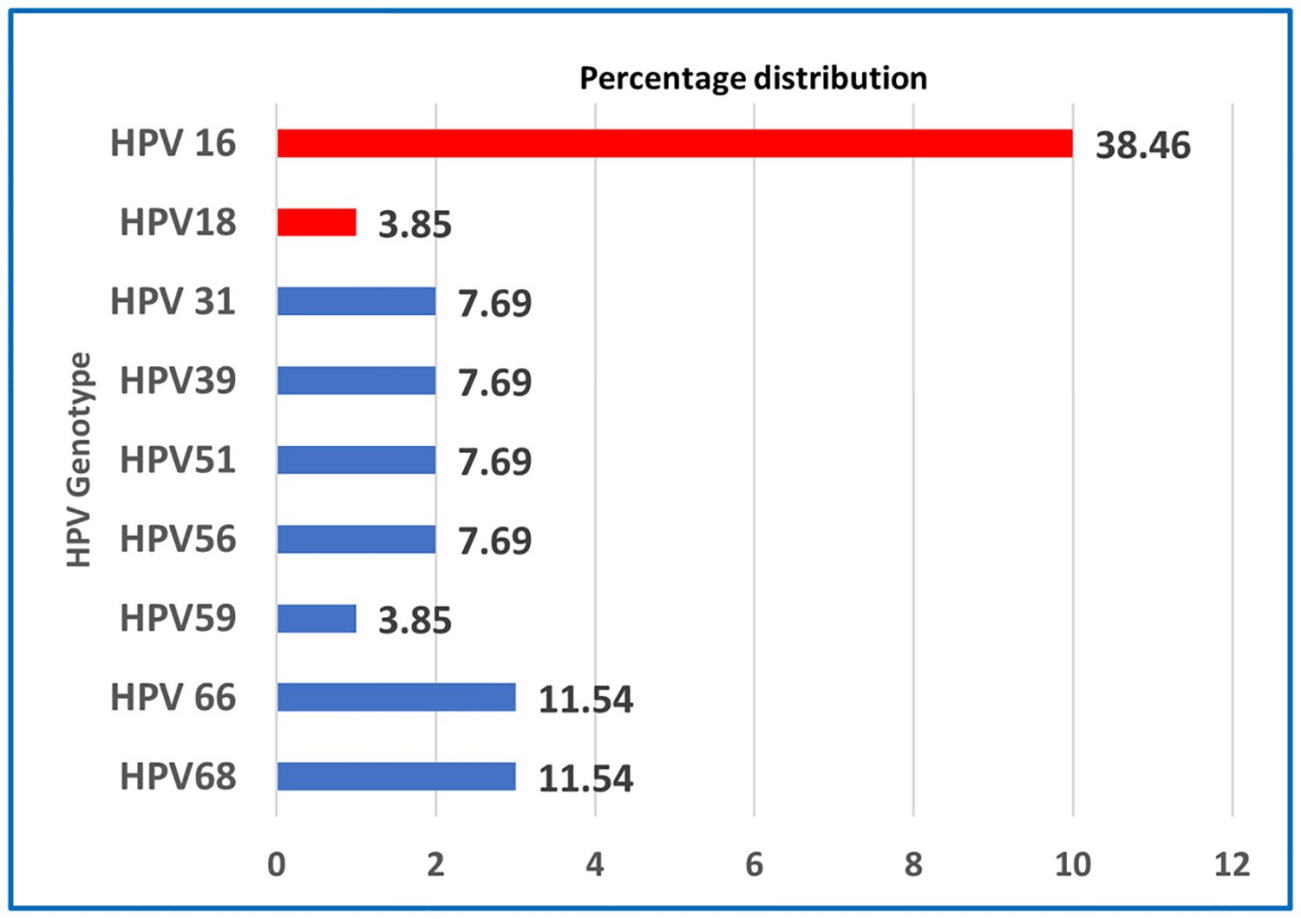
Individual distribution of HR-HPV genotype among participants (n = 26).

Individual distribution of HR-HPV genotype showed HPV 16 was the most prevalent genotype, consisting 38.46% (n = 10) of the total positive samples followed by HPV 66 and HPV 68 both contributes 11.54% each. However, HPV 31, HPV 39, HPV 51, and HPV56 each contributes 7.69% [Fig 2].

**Fig 3:**
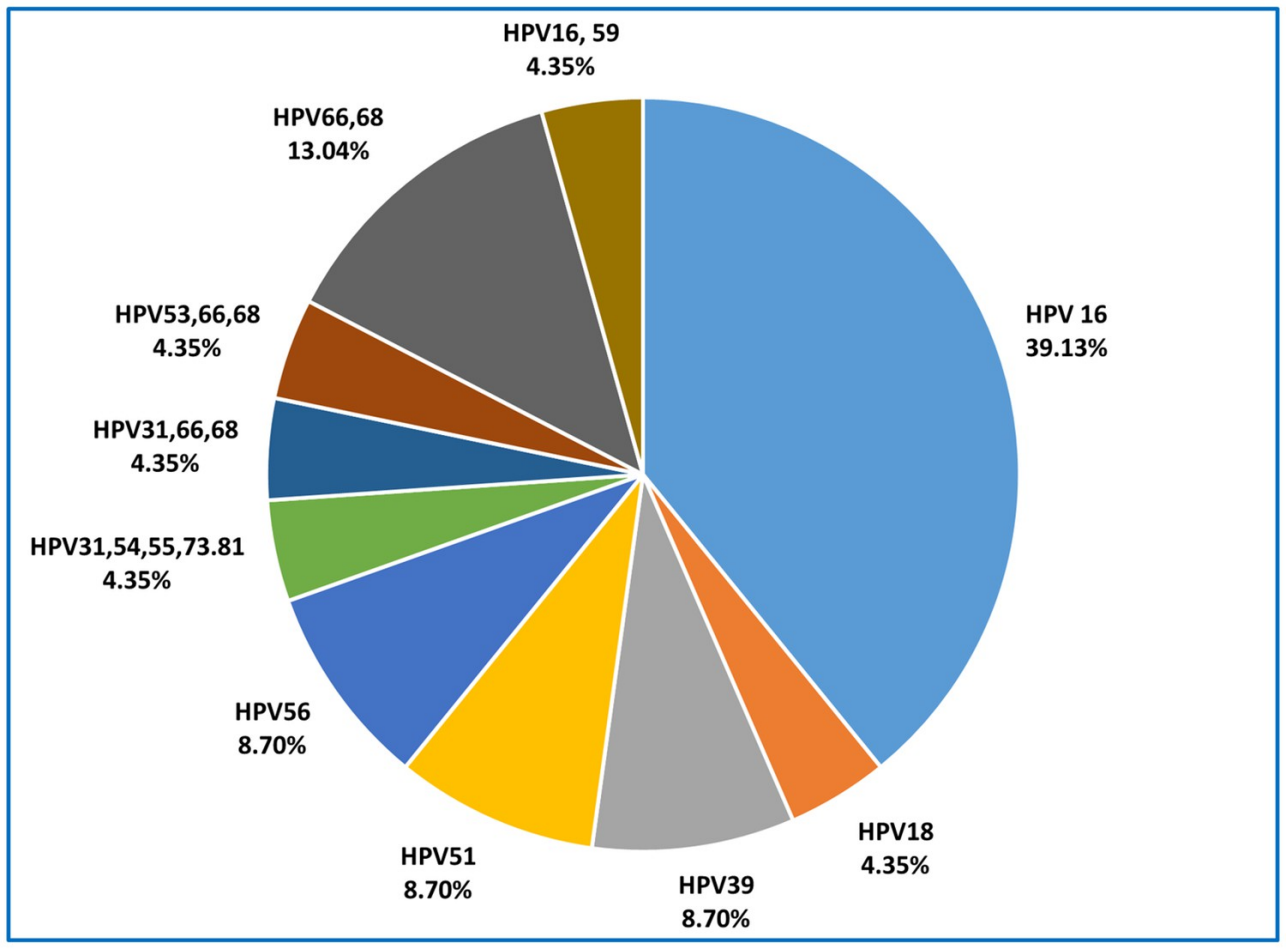
HR-HPV positive women with distribution with single and multiple infections (n = 23).

Furthermore, when considering distribution as single and multiple genotypes showed single infection by HPV 16 was the most prevalent infection (39.13%) followed by multiple infection by HPV66,68 (13.04%). However, single infection by HPV 39, HPV51 and HPV 56 contributes 8.70% for each. HPV 18 contributes only in one women (4.35%) **[**Fig 3].

### Association of HR-HPV DNA with socio-demographic and reproductive factors

HR-HPV prevalence across different age groups illustrated an increase in positivity with age, ranging from 1.1% in age 30–34 year with the highest positivity rate observed in the 55–60 years’ of age group (5.5%). Whereas only exception was age group 50–54 year which showed declined positivity (1.9%). Despite this trend, statistical testing across age groups did not reveal significant differences (p-value = 0.40). The HR-HPV positivity was higher among women with no formal education (4.0%), among women whose husband with no formal education (5.6%), among housewife (2.8%) and women with monthly income 5001–10000 BDT (4.1%) [Table 1]. However, none of these factors had statistically significant (p>0.05) association with HPV positivity. Furthermore, Women who were married at or before 15 years of age (4.2%) and had their first delivery at or before 15 years of age (3.1%), primiparous (3.2%) and husband living in different place (7.1%) had higher HPV positivity [Table 2]. However, none of these factors had statistically significant association with HPV positivity (P>0.05).

**Table 2:** Association of reproductive variables with HR-HPV result of study participants (n = 900).

Variables	HR-HPV Positive N(%)	HR-HPV Negative N(%)	p-value *
**Age at Marriage (years)**			
≤ 15	11(4.2)	251(95.8)	0.06 *
16–17	2(0.9)	228(99.1)	
≥18	10(2.5)	398(97.5)	
**Age at First delivery (years) (n = 884)**			
≤ 15	2(3.1)	62(96.9)	0.91*
16–20	14(2.6)	518(97.4)	
21–25	6(2.8)	208(97.2)	
≥26	1(1.4)	73(98.6)	
**Parity**			
Nuliparous	0(0)	17(100)	0.58**
Primiparous	2(3.2)	61(96.8)	
Multiparous	21(2.6)	799(97.4)	
**Husbands Living Status**			
Living Together	20(2.5)	781(97.5)	0.54**
Living in different place	2(7.1)	26(92.9)	
Aborad	0(0)	24(100)	
Dead	1(2.3)	42(97.7)	
Divorced	0(0)	4(100)	

Chi-square test = *, Fisher’s Exact tests = **

### HPV prevalence and genotypes by geographic location

The prevalence of HPV among different coastal districts [Table 3] showed Jhalkhati had the highest prevalence (3.0%), followed by Cox’s bazar (2.7%), and Bagerhat (2.0%). This variation was non-significant (p-value = 0.73). Among 23 participants tested positive for HPV, HPV 16 was the most prevalent genotype, detected in 9 participants as single infection. HPV 16 was dominant genotypes in all three districts. However only one HPV 18 was found in Bagerhat. HR-HPV genotypes distribution were varied among districts non significantly (P >0.05) [Table 3].

**Table 3:** Distribution of participant HPV genotype by districts n = 23.

HR-HPV Genotype	District Name				
	Jhalokathi	Cox’s Bazar	Bagerhat	Total	P-value
**HPV 16**	4 (44.4%)	3 (37.5%)	2 (33.3%)	9 (39.1%)	
**HPV 18**	0 (0.0%)	0 (0.0%)	1 (16.7%)	1 (4.3%)	0.62^**^
**HPV 39**	1 (11.1%)	0 (0.0%)	1 (16.7%)	2 (8.7%)	
**HPV 51**	1 (11.1%)	1 (12.5%)	0 (0.0%)	2 (8.7%)	
**HPV 56**	1 (11.1%)	1 (12.5%)	0 (0.0%)	2 (8.7%)	
**HPV 31, 54, 55, 73, 81**	1 (11.1%)	0 (0.0%)	0 (0.0%)	1 (4.3%)	
**HPV 31, 66, 68**	1 (11.1%)	0 (0.0%)	0 (0.0%)	1 (4.3%)	
**HPV 53, 66, 68**	0 (0.0%)	1 (12.5%)	0 (0.0%)	1 (4.3%)	
**HPV 66, 68**	0 (0.0%)	1 (12.5%)	2 (33.3%)	3 (13.0%)	
**HPV 16, 59**	0 (0.0%)	1 (12.5%)	0 (0.0%)	1 (4.3%)	
**Total**	9 (100%)	8 (100%)	6 (100%)	23 (100%)	

Fisher’s Exact tests = **

## Discussion

Limited research has been conducted on the prevalence of HR-HPV infections in Bangladesh, and this study represents the first investigation to specifically examine the prevalence and genotype distribution of HR-HPV among ever-married women residing in the coastal regions of the country. The study revealed that the overall prevalence of HR-HPV among the study population was 2.56%. This prevalence was lower compared to a population-based study among asymptomatic women in Dhaka division of Bangladesh with 7.7% overall HPV prevalence and 4.2% of HR-HPV prevalence [19]. Nonetheless, the prevalence of HR-HPV genotypes varies across various populations [19, 23–26]. A study conducted in South-coastal Karnataka, southern India, found higher cervical HR-HPV infection (10.5%) than coastal women of Bangladesh [27]. Overall low socio-economic status was mentioned as related factor for high cervical HR-HPV infection and both sexual and non-sexual factors including hygiene and nutritional deficiencies might be related. Also among the coastal women of Da Nang, Vietnam city, an overall prevalence of HR-HPV genotype was also high (9.5%) [28]. A cohort study among Tunisian women showed higher HR-HPV prevalence (14.9%) even among women with normal cytology [29]. Worldwide overall HR-HR-HPV prevalence in women with normal cytology at any given point of time is approximately 11–12% indicating that HPV is one of the most common sexually transmitted infections [30]. Therefore, the HR-HPV prevalence among Bangladeshi women particularly in the coastal areas was lower than many parts of the world. This may be related to geographical, social and cultural factors, variation of sexual behaviour due to more conservative norms and religious traditions and practice of male circumcision [31, 32].

The non-significant statistical differences observed in HR-HPV prevalence among the three districts may potentially indicate variations in regional health behaviors, screening practices, or healthcare accessibility. The notably higher prevalence of HR-HPV in Cox’s Bazar district suggests the need for targeted interventions or a comprehensive investigation to elucidate the underlying factors contributing to this disparity. In the present study, when genotype is considered among the HR-HPV positive cases as individual involvement, HPV16 is the most common genotype found among women living in coastal areas (38.46%), followed by HPV 66 and HPV 68 (11.54% each). Additionally, HPV 31, HPV 39, HPV 51, and HPV 56 each accounted for 7.69% of positive samples. This highlights the diversity of HR-HPV genotypes circulating in the study population. While considering as single and mixed infection, HPV 16 contributed 39.13% followed by HPV 66,68 (13.04%), HPV56(8.70%), HPV51(8.70%) and HPV 39(8.70%) with HPV 18 had a very low prevalence. It is noteworthy to observe that Nahar’s study conducted in the Dhaka division of Bangladesh yielded similar results, with HPV16 and HPV66 being the most frequently detected high-risk HPV (HR-HPV) types followed by HPV18, HPV45, HPV31, and HPV53 [19]. The present study revealed HPV 66, 68 as more prevalent genotypes compared to low prevalence of HPV18. However, several studies in Asian women mentioned that HPV 52 and HPV 58 as more prevalent genotypes associated with the development of CC [33, 34].

Fujian in the southeastern coast of China had a high prevalence of HR-HPV infection (33.9%), with most prevalent HR-HPV genotype as HPV 16 (8.5%), followed by HPV 52 (7.9%), HPV 58 (6.2%), and HPV 53 (3.5%), and a relatively low prevalence of HPV 18 [35]. Remarkably, the absence of HPV52 and HPV58 was observed among women residing in the coastal areas of Bangladesh, despite their low prevalence in the Dhaka division. Consequently, among coastal regions, HPV16, HPV66, and HPV68 emerge as the three most prevalent genotypes among women, displaying similarities to the Dhaka division where high prevalence of HPV16 and HPV66 is observed. It is noteworthy that HPV 66 and HPV 68 were also prevalent among the coastal population of Da Nang, Vietnam [28]. However, the HPV16 was consistently the most common type and HPV 18 as the second common infection in women with normal cytology globally with minor regional differences [36].

The HR-HPV prevalence across different age groups showed an increasing trend with age, peaking among women aged 55–60 years, with a slight decline observed in the 50-54-year age group. Similarly, factors such as lack of formal education in women and their husbands, being a housewife, lower monthly income, early marriage and childbirth, and having a husband living in a different location were linked to higher HR-HPV positivity rates. However, these associations were not statistically significant. These findings suggest that while certain demographic and reproductive factors may influence HPV prevalence trends, they may not independently determine HR-HPV infection risk in this population. The increase in HPV positivity with age contradicts some studies suggesting a peak in younger populations, possibly reflecting persistent infections leading to higher risk of cervical pathology among older women. This trend, despite not being statistically significant, points to the importance of CC screening across higher age groups.

Primary HPV screening with HPV16/18 partial genotyping is a promising approach to recognize women at risk of >CIN2 and this reduces referral of women for colposcopy [34, 36]. Therefore, adoption of HPV test for CC screening in this population is important as more prevention can be achieved with less referral. The Portland Cohort Study also strongly supported for HPV testing due to the high negative predictive value and the extremely low risk of CIN3 or cancer following a negative HPV test in women ≥30, lasting up to 15+ years [37]. Therefore adoption of HPV test is more applicable for coastal areas of Bangladesh with high prevalence of HPV16. Even though the HR-HPV prevalence is comparatively low, once in a lifetime screening after the age of 30 along with treatment will certainly reduce the risk of CC in this neglected population. Though the national screening programme is continuing a VIA-based programme, the GOB need to consider HPV-based screening in the coastal areas. However, challenge remained regarding management of the HR-HPV positive women as colposcopy clinics are available mostly at the district level. GOB need to develop Thermal Ablation (TA) facilities at the sub-district level at the costal areas where VIA trained nurses can provide treatment services and all HPV16 women should be offered treatment by TA considering eligibility. However, HPV 16/18 positive women not eligible for TA should be navigated to the colposcopy clinic with LEEP facility. As the women of costal areas live in hard to reach areas with difficulties to attend colposcopy clinics for follow-up and repeat HR-HPV test, ‘Other HPV types’ should be treated also as they are in high number and loss to follow-up of this group may lead to development of CC in some of the cases.

The GOB has introduced bivalent HPV vaccine (targets HPV 16, 18) in the national immunization programme in October, 2023 [38]. Considering our GOB should have considered a vaccine that covers more HR-HPV types.

In the coastal areas, 45.00 million (26.50% of the total population) resides and among them presently 6,232,770 women (21.48%) of the total target women (30–60 Years) need cervical screening [39]. The strong tidal waves from the Indian Ocean through the Bay of Bengal continuously hit the land of the delta causing erosion to keep the coastal areas unstable [40]. Moreover, the dynamic coastal environment of Bangladesh, characterized by intense monsoon rainfall, rising sea levels, and coastal erosion, poses significant public health challenges. These environmental factors may contribute to the unique patterns of HR-HPV prevalence observed in the coastal populations [41, 42]. The vulnerability in the coastal population of Bangladesh is even more due to geographic settings, dense population, and poverty. Therefore, formulation of effective vaccination and screening programme is necessary for this significant number of susceptible population living in hard to reach areas with poorly developed health facilities. This finding of this study should encourage policy makers for resigning vaccine arrangement policies, with importance of the need for vaccines specific for this regions.

This study includes women of 30–60 years of age attending to health facilities to avail screening facilities. The limits the generalizability of the study. This study could not complete the colposcopic and management data of the HR-HPV positive individuals, which might provide baseline information on cervical lesions.

## Conclusion and recommendation

The cross-sectional study reveals a relatively low prevalence of HR-HPV infections among women in coastal regions. Though HPV 16 is most common, HPV 66, HPV 66 forms a second common group and HPV18 was comparatively rare infection. The currently available bivalent vaccine may have lower efficiency among coastal Bangladeshi women.

Introduction of screening by HR-HPV genotyping, integration of HPV vaccination considering genotype distribution and remodeling of management of Other HR-HPV infections for Costal areas are recommended for combating CC in this zone.
